# *TFEB* ‐translocated and ‐amplified renal cell carcinoma with *VEGFA* co‐amplification: A case of long‐term control by multimodal therapy including a vascular endothelial growth factor‐receptor inhibitor

**DOI:** 10.1002/iju5.12575

**Published:** 2023-03-01

**Authors:** Hajime Takamori, Akiko Miyagi Maeshima, Ikuma Kato, Masaya Baba, Eijiro Nakamura, Yoshiyuki Matsui

**Affiliations:** ^1^ Department of Urology National Cancer Center Hospital Tokyo Japan; ^2^ Department of Diagnositic Pathology National Cancer Center Hospital Tokyo Japan; ^3^ Department of Molecular Pathology Yokohama City University Graduate School of Medicine Yokohama Japan; ^4^ International Research Center for Medical Sciences Kumamoto University Kumamoto Japan

**Keywords:** carcinoma, renal cell, gene amplification, in situ hybridization, fluorescence, transcription factors, vascular endothelial growth factor A

## Abstract

**Introduction:**

Renal cell carcinoma with *TFEB* amplification is rare and reportedly aggressive. We herein report a case of renal cell carcinoma with *TFEB* translocation and amplification in which long‐term control was achieved by multimodal therapy including a vascular endothelial growth factor ‐receptor inhibitor.

**Case presentation:**

A 70‐year‐old man was referred to our institution for the treatment of renal cell carcinoma with multinodal metastases. Open nephrectomy and lymph node dissection were performed. Immunohistochemistry for transcription factor EB was positive, and fluorescent in situ hybridization revealed *TFEB* rearrangement and amplification. The diagnosis was *TFEB*‐translocated and ‐amplified renal cell carcinoma. *VEGFA* amplification was also demonstrated by fluorescent in situ hybridization. The residual and recurrent tumors were treated and controlled for 52 months by vascular endothelial growth factor‐receptor target therapy, radiation therapy, and additional surgery.

**Conclusion:**

A good long‐term response to anti‐vascular endothelial growth factor drug therapy may be due to *VEGFA* amplification and subsequent vascular endothelial growth factor overexpression.

**Abbreviations & Acronyms**
CT: computed tomography
FISH: fluorescence in situ hybridization
RCC: renal cell carcinoma
TFEB: transcription factor EB
VEGFA: vascular endothelial growth factor A
VEGFR: vascular endothelial growth factor‐receptor
WHO: World Health Organization

**Keynote message**

*VEGFA* has been reported to be amplified in *TFEB*‐amplified RCC. VEGF may be a potential therapeutic target in *TFEB*‐amplified RCC.

## Introduction

In the 2016 WHO classification, *TFEB*‐translocated RCC first emerged. In the 2022 WHO classification, rarer *TFEB*‐amplified RCC was first included in the “*TFEB*‐altered RCCs” group.^1^ Some investigators have reported its aggressive nature and concurrent *VEGFA* gene amplification.^2^ We herein report a case of *TFEB*‐translocated and ‐amplified RCC for which we achieved long‐term control by multimodal therapy, including a VEGFR inhibitor.

## Case presentation

**Fig. 1:**
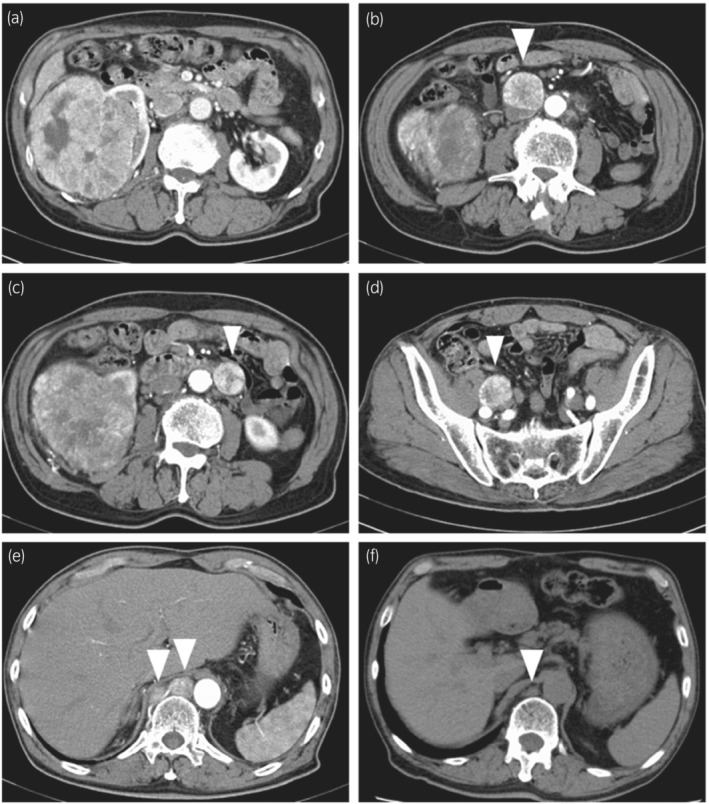
Abdominal contrast‐enhanced computed tomography at the initial diagnosis. Imaging revealed (a) a 12‐cm renal tumor in the right kidney and multinodal metastases in the (b) inter‐aortocaval, (c) para‐aortic, (d) external iliac, and (e) posterior mediastinal regions. (f) Abdominal plain computed tomography 1 year after administration of axitinib shows shrinkage of the posterior mediastinal lesions.

**Fig. 2:**
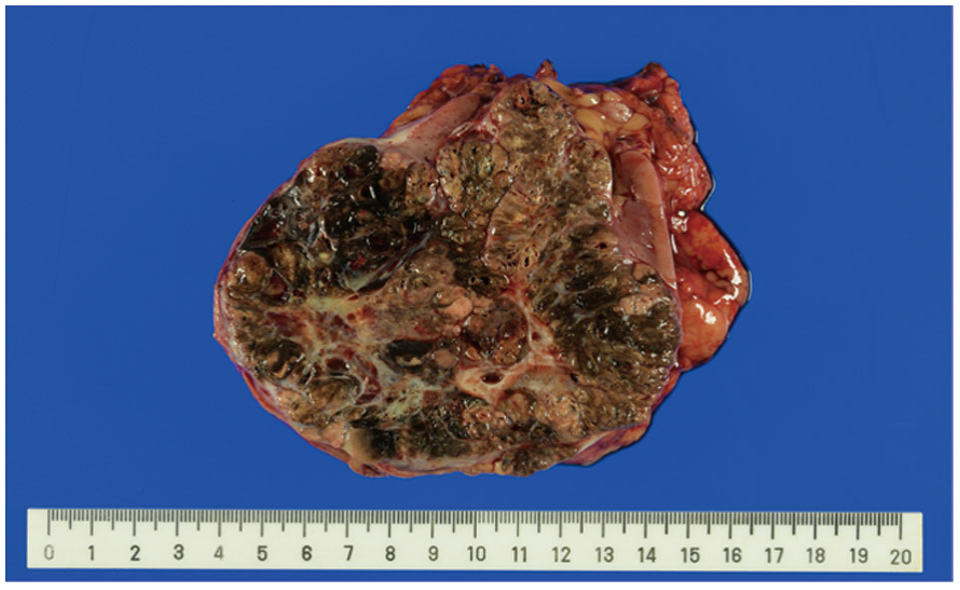
Gross appearance of the resected renal tumor. The cut surface was black‐brown in color.

**Fig. 3:**
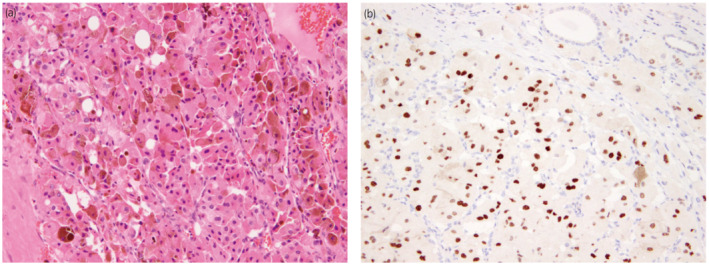
Microscopic findings. (a) Hematoxylin and eosin staining revealed a solid, partly cystic structure of tumor cells with large eosinophilic cytoplasm containing abundant melanin. (b) Immunohistochemical staining was positive for transcription factor EB.

**Fig. 4:**
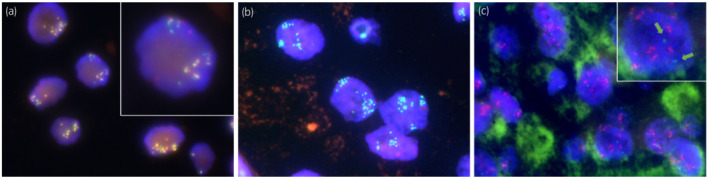
Fluorescence in situ hybridization (FISH). (a) FISH (*TFEB* break apart) showing *TFEB* gene rearrangement and amplification. The green and red signals are split apart, demonstrating the break of the *TFEB* gene (insert). The remarkably increased number of signals indicates the amplification of *TFEB*. (b) Fusion probe FISH shows that *MALAT1* (red) was not a fusion partner of *TFEB* (green). Amplification of *TFEB* was confirmed. (c) FISH shows an identical level of amplification of *VEGFA*. Highly increased fluorescent signals of *VEGFA* (red) per neoplastic nucleus were observed, whereas two signals of the Chromosome 6 Control Probe (green) were observed per nucleus (green arrows in the inserted figure), indicating *VEGFA* gene amplification without chromosomal amplification.

A 70‐year‐old man was referred to our institution for treatment of a right kidney tumor. The tumor was incidentally detected on a computed tomography (CT) scan. The CT scan (Fig. 1a–e) revealed a right kidney tumor with a maximum diameter of 12 cm accompanied by lymph adenopathy in the inter‐aortocaval, para‐aortic, external iliac, and mediastinal regions. No visceral metastasis was observed. The clinical diagnosis was metastatic RCC. Open nephrectomy and lymph node dissection were performed. The mediastinal lesions were not resected because of the difficulty in approaching them. Macroscopically, a brown‐black tumor measuring 12 × 10 × 8 cm showed expansive growth (Fig. 2). Microscopic examination revealed a solid, partly cystic structure of tumor cells with large eosinophilic cytoplasm containing abundant melanin (Fig. 3a). Immunohistochemistry was positive for AE1/3, PAX8, and melan A and negative for alpha‐smooth muscle actin. Therefore, melanoma and epithelioid angiomyolipoma were ruled out. We suspected MiT family translocation RCC as melanin‐producing RCC. Immunohistochemistry was positive for TFEB (Fig. 3b) and negative for TFE3. FISH using a *TFEB* split probe (GSP Lab., Inc., Kobe, Japan) was carried out to attain a definitive diagnosis (Fig. 4a). *TFEB* rearrangement was confirmed. Additionally, *TFEB* gene signals were remarkably increased, indicating *TFEB* amplification. FISH using a *MALAT1*‐*TFEB* fusion probe (GSP Lab., Inc.) did not demonstrate *MALAT1*‐*TFEB* gene fusion (Fig. 4b). Based on the above results, although the *TFEB* fusion partner gene was not identified, the diagnosis was *TFEB*‐translocated and ‐amplified RCC. In addition, FISH using a *VEGFA* probe and the Chromosome 6 Control Probe (Empire Genomics, Buffalo, NY, USA) was performed (Fig. 4c). The results indicated *VEGFA* amplification without chromosomal amplification.

Because the mediastinal lesions remained unresected, pazopanib was administered. We did not select a combination of tyrosine kinase inhibitor and immunotherapy because they were not standard at that time. Two weeks after administration, pazopanib was discontinued because of liver dysfunction, and axitinib was started. Immediate shrinkage of the lesions was observed (Fig. 1f). Two years after administration of axitinib, positron emission tomography–CT revealed no new lesions. Radiation therapy was performed on the mediastinal lesions, and axitinib was discontinued. Six months later, a metastatic lesion was found in the para‐aortic region. We performed laparoscopic resection, and the microscopic findings revealed almost the same tissue as the primary tumor. Three months later, a CT scan revealed new presacral lesions. After re‐administration of axitinib, shrinkage of the presacral lesions was observed. However, liver metastasis was noted on a CT scan 18 months after axitinib re‐administration, and nivolumab was initiated. Nevertheless, the disease was controlled for 52 months by multimodal treatment.

## Discussion

In this report, we presented a case of *TFEB‐*translocated and ‐amplified RCC with multiple lymph node metastases. We controlled the disease for 52 months by multimodal therapy including surgeries, a VEGFR inhibitor, and radiation therapy.

MiT family translocation RCC is a newly introduced category of renal neoplasia in the 2016 WHO classification.^1^ *TFEB* translocation RCC is a type of MiT family translocation RCC; it harbors gene fusions involving *TFEB*, resulting in overexpression of native TFEB. *TFEB* translocation RCC is rare (0.02% of all renal carcinomas). The mean age of affected patients is 34 years, with a wide reported range of 3 to 77 years. There is no remarkable sex predominance (female:male ratio, 0.75:1.00).^3^ Most cases of *TFEB* translocation RCC reportedly have an indolent clinical course.^4^ The *TFEB* is located in the short arm of chromosome 6, specifically in the 6p21‐p23 region.^5^ The most commonly reported fusion partner for the *TFEB* is the *MALAT1* (81%),^4^ and more recent studies have revealed *ACTB*, *NEAT1*,^6^ *COL21A1*, and *CADM2* ^7^ as rarer fusion partners. In the present case, the fusion partner and the exact fusion point could have been identified by reverse‐transcription polymerase chain reaction. Unfortunately, we did not store frozen specimens. The MiT family of transcription factors is involved in melanocyte and osteoclast differentiation.^8^ Immunohistochemical stains for melanocyte‐related antigens and osteoclast markers play supportive roles in the diagnosis of MiT family translocation RCCs.^9^

*TFEB*‐amplified RCC was first included in the “*TFEB*‐altered RCCs” group in the 2022 WHO classification.^1^ This type of RCC is extremely rare, with only approximately 60 reported cases worldwide to date.^9^ *TFEB*‐amplified RCC differs from unamplified *TFEB* translocation RCC in several ways.^2^ First, the mean age at presentation is 65 years, which is higher than that at the presentation of *TFEB* translocation RCC. Second, its morphology is usually highly malignant in terms of the International Society of Urologic Pathologists/WHO 2016 nucleolar grade. Finally, patients with *TFEB*‐amplified RCC typically have an aggressive clinical course. Concurrent *VEGFA* amplification has been reported in *TFEB*‐amplified RCC.^2^, ^10^ In addition, frequent expression of programmed death‐1 ligand 1 was described in both *TFEB* translocation RCC^6^ and *TFEB*‐amplified RCC.^11^

In the present case, not only *TFEB* but also *VEGFA* was amplified, as in the previously reported cases. *VEGFA* is immediately adjacent to *TFEB* at 6p21.1. Given the proximity of these two genes, the *VEGFA* status has been investigated in *TFEB*‐amplified RCC.^12^ In a study of RCC with *TFEB* gene alternation, Caliò et al.^10^ stated that *VEGFA* amplification is considered to lead to overexpression of VEGF and enhancement of angiogenesis and that VEGF is a potential therapeutic target for *TFEB*‐amplified RCC. Likewise, in the present case, we assumed that VEGF was overexpressed. We hypothesized that the good long‐term response to the VEGFR inhibitor was due to VEGF overexpression. Further investigations are warranted to determine whether patients with *TFEB*‐amplified RCC are good responders to VEGFR target therapy.

## Conclusion

We have herein presented a case of *TFEB*‐translocated and ‐amplified RCC with multinodal metastases that were controlled for 52 months by multimodal treatment. The good response to the VEGFR inhibitor may have been due to the concurrent amplification of *VEGFA* and subsequent VEGF overexpression.

## Author contributions

**Hajime Takamori:** Conceptualization; formal analysis; investigation; writing – original draft. **Akiko Miyagi Maeshima:** Data curation; formal analysis; writing – review and editing. **Ikuma Kato:** Data curation; writing – review and editing. **Masaya Baba:** Data curation; writing – review and editing. **Eijiro Nakamura:** Conceptualization; project administration; writing – review and editing. **Yoshiyuki Matsui:** Project administration, supervision, writing – review and editing.

## Conflict of interest

The authors declare no conflict of interest.

## Approval of the research protocol by an Institutional Reviewer Board

This study was approved by the Institutional Review Board of the National Cancer Center Hospital (study approval number: 2017‐168).

## Informed consent

Written informed consent was obtained from the patient.

## Registry and the registration No. of the study/trial

Not applicable.
